# Editorial: Women in animal behavior and welfare: 2021

**DOI:** 10.3389/fvets.2022.1106052

**Published:** 2022-12-15

**Authors:** Gabriella Guelfi, Nicole Kemper

**Affiliations:** ^1^Department of Veterinary Medicine, Università degli Studi di Perugia, Perugia, Italy; ^2^Institute for Animal Hygiene, Animal Welfare and Farm Animal Behaviour, University of Veterinary Medicine Hannover, Hannover, Germany

**Keywords:** animal welfare, animal behavior, epigenetics, female scientists, human care, stress, living environment

The work of female scientists often does not receive the attention it deserves. To recognize and promote the achievements of women in animal behavior and welfare science, this Research Topic aims to highlight the scientific contributions of women researchers in this area. In animal behavior and welfare science, as a relatively young field, which gained increasing scientific importance just over the last two decades, the proportion of female researchers is at a high level already. However, no documented numbers regarding gender balance exist.

With this Research Topic, we honor the merits of female scientists in animal behavior and welfare science, a field of great public interest that is increasing even still. The subject of this scientific field is the mental and physical state of an animal while interacting with its living environment (i.e., health, care, stress, feeding and supplemental feeding, learning and stimuli enhancement). Traditional methods such as animal behavior assessment via direct observations have been technically improved over the last decade by up-to-date techniques such as advanced video recordings and automatic analyses of behavioral traits and complete ethograms. Another groundbreaking aspect is the new multi-omics profiling approach, the existence of molecular mechanisms allowing genotype-environment interactions, the so-called epigenetic mechanisms. According to this theory, the living environment selects which gene has to be turned on and which one to be turned off. Transferred to animal welfare, this means that positive environmental stimuli guarantee animal welfare, while negative stimuli predispose to the onset of various diseases (1). The epigenetic mechanism as a final effect has an impact on the animal phenotype, and, therefore, on its welfare and the development of behavior (2–4).

The variety of different scientific methodologies and approaches to evaluate and improve animal welfare in different species is presented in the contributions to this Research Topic. The papers do not only consider the most relevant livestock species but deal with other animals kept for human use, for instance as laboratory animals such as zebrafish, as in the research carried out by Leyden et al. In their study, the impact of tricaine, the most commonly used chemical anesthetic in zebrafish research, on different physiological parameters is thoroughly evaluated and compared to gradual cooling. New insights were generated, but the results also clearly show the need for further research regarding the potential of appropriate alternative anesthetic agents for the sake of zebrafish welfare.

Staying with aquatic species, but concentrating on invertebrates, Wahltinez et al. provide a comprehensive overview of this hitherto neglected topic in their perspective article. Aquatic invertebrates, such as cephalopod mollusks and decapod crustaceans, can suffer stress and feel pain, too. The authors encourage the protection of aquatic invertebrate welfare and provide practical recommendations using anesthesia, analgesia, and euthanasia in addition to non-invasive handling methods in aquaculture and fisheries. With this important contribution, the authors advocate further research in this underrepresented but important field of animal welfare.

Changing to poultry, one major welfare issue in laying hens is feather pecking, often followed by cannibalism. One prevention measure is the provision of an adapted feeding regime with supplements. In their study, Mindus et al. analyze the impact of dietary supplementation with *Lactobacillus rhamnosus* JB-1 probiotic bacteria against stress-induced severe feather pecking damage. Based on their results, the authors suggest that this probiotic strain may have beneficial effects on the avian immune response and the prevention of feather pecking and plumage damage, thus increasing animal health and welfare.

Junghans et al. provide an exploratory study on the evaluation of fattening and slaughtering of broiler chickens by multivariate analyses, considering different factors comprehensively. Several factors were identified that can affect the mortality of broilers during the rearing period, their slaughter weight, and the causes of condemnation recorded at the processing plant. With these new insights, the authors show the potential of minimizing the use of antibiotics on farms where animal welfare is ensured.

Comprehensive statistical analyses on the base of a large data set were also the basis of the research presented by Dachrodt et al. They not only give a detailed overview of the status quo of colostrum, feeding, and housing practices of pre-weaned dairy calves in German dairy farms but also developed a benchmark system to evaluate calf health on farms and to identify potential problem areas. For all persons involved in calf management, such as farmers, herd managers, veterinarians, and other advisors, this tool is beneficial to assess on-farm calf health and thus brings this topic, for the benefit of calves' welfare, into focus.

On a more experimental level, Stenfelt et al. explored whether dairy cows have the cognitive abilities to learn new behavior *via* social learning. In their experiments, they showed that cows did not utilize social learning mechanisms when solving a spatial detour task. The knowledge of social learning in farm animals is very limited, and with these new insights, the authors provide essential new information and open the space for further research questions concerning the cognitive abilities of cattle.

Other milk-producing species, more precisely sheep and goats, are present in this Research Topic, too. The study carried out by Berthel et al. describes the preference of non-lactating dairy sheep and goats for a diet containing a monocomponent vs. a mixed ration of the same components and similar nutritional value. This new aspect can be used in creating adapted diets, considering ruminants' natural behavior of selective feeding, and improving their wellbeing in that way.

To close the circle of animal species, Carroll et al. present a study evaluating the prevalence of adoption and relinquishment of dogs and cats during the COVID-19 pandemic. They identified risk factors for relinquishment and put, with this innovative study, the topic into focus. Especially for information on prevention and interventions aiming at the reduction of companion animal relinquishment, these findings are of utmost importance.

Finally, in the review article by Krebs et al., the influences of space, time, and context on patterns of anticipatory behaviors in animals under human care are discussed intensively. Unidentified anticipation can alter conclusions regarding animal behavior or welfare under certain circumstances, and the authors explain for instance, how animals are driven to anticipatory behavioral models by reward desire. With this work, valuable advice is given on how such impairments in animal welfare research can be identified and taken into account.

Concluding, the body of research included in this Research Topic impressively shows the various contributions female scientists bring to the field of animal behavior and welfare research. By providing science-based results to increase the knowledge of the effects of the living environment on animal welfare and behavior, useful, practical approaches to improve the welfare of a variety of species kept and used by humans can be derived. These improvements rest in large part on the shoulders of female scientists, working on basic and applied research projects now and in the future for the benefit of the animals.

## Author contributions

All authors listed have made a substantial, direct, and intellectual contribution to the work and approved it for publication.
